# Changes in the Transcriptome and Chromatin Landscape in BRAFi-Resistant Melanoma Cells

**DOI:** 10.3389/fonc.2022.937831

**Published:** 2022-06-17

**Authors:** Kiran Kumar Reddi, Praveen Guruvaiah, Yvonne J. K. Edwards, Romi Gupta

**Affiliations:** ^1^ Department of Biochemistry and Molecular Genetics, The University of Alabama at Birmingham, Birmingham, AL, United States; ^2^ O’Neal Comprehensive Cancer Center, The University of Alabama at Birmingham, Birmingham, AL, United States

**Keywords:** MAPK, melanoma, BRAF, MEK, transcriptomics, chromatin accessibility

## Abstract

Metastatic and drug-resistant melanoma are leading causes of skin cancer–associated death. Mitogen-associated protein kinase (MAPK) pathway inhibitors and immunotherapies have provided substantial benefits to patients with melanoma. However, long-term therapeutic efficacy has been limited due to emergence of treatment resistance. Despite the identification of several molecular mechanisms underlying the development of resistant phenotypes, significant progress has still not been made toward the effective treatment of drug-resistant melanoma. Therefore, the identification of new targets and mechanisms driving drug resistance in melanoma represents an unmet medical need. In this study, we performed unbiased RNA-sequencing (RNA-seq) and assay for transposase-accessible chromatin with sequencing (ATAC-seq) to identify new targets and mechanisms that drive resistance to MAPK pathway inhibitors targeting BRAF and MAPK kinase (MEK) in *BRAF*-mutant melanoma cells. An integrative analysis of ATAC-seq combined with RNA-seq showed that global changes in chromatin accessibility affected the mRNA expression levels of several known and novel genes, which consequently modulated multiple oncogenic signaling pathways to promote resistance to MAPK pathway inhibitors in melanoma cells. Many of these genes were also associated with prognosis predictions in melanoma patients. This study resulted in the identification of new genes and signaling pathways that might be targeted to treat MEK or BRAF inhibitors resistant melanoma patients. The present study applied new and advanced approaches to identify unique changes in chromatin accessibility regions that modulate gene expression associated with pathways to promote the development of resistance to MAPK pathway inhibitors.

## Introduction

Melanoma is the deadliest form of skin cancer, and aggressive or drug-resistant forms of melanoma can metastasize to various distal organs (1, 2). Genomic sequencing has identified oncogenic *BRAF* mutations in greater than 50% of melanoma tumors (3, 4). Acquired oncogenic *BRAF* mutations result in the constitutive activation of BRAF→MEK→ERK (MAPK) pathway, which is necessary for melanoma growth and progression (5–7). These findings have led to the development of several BRAF and MEK inhibitors (BRAFi and MEKi, respectively) that have received approval by the US Food and Drug Administration (FDA) for use in the treatment of unresectable metastatic melanoma (8–10). However, despite an initial robust response to BRAF- and MEK-targeted therapies, patients with melanoma typically acquire treatment resistance within a few months, resulting in disease progression (11, 12). Due to the high prevalence of acquired resistance to BRAFi and MEKi, intensive efforts have focused on identifying the underlying mechanisms (4, 12, 13), contributing to substantial progress in the treatment of advanced and drug-resistant melanoma (14, 15). However, in a subset of cases, the mechanisms underlying acquired BRAFi and MEKi resistance remain unknown, and continued efforts toward identifying the drivers of treatment resistance in these cases remain necessary to identify more efficient, durable, and potentially personalized treatment options.

Mechanism of resistance to BRAF/MEK inhibitors can be MAP kinase pathway dependent and independent. The goal of our study was to investigate the role of MAP kinase pathway independent- reprogramming of chromatin landscape in MAPK pathway inhibitor resistant melanoma cells. Here, we focused on identifying alterations in genomic distribution of accessible chromatin site and its impact on transcriptional network to identify the functional genes and pathways that can be targeted for the treatment of MAPK pathway inhibitor resistant melanoma. To do so, in this study we performed deep-sequencing analyses in BRAFi resistant and sensitive melanoma cells, after confirming their growth phenotype. Our results revealed that the combined use of MAPK pathway inhibitors significantly inhibited the growth of BRAFi-sensitive cells but had only modest effects on BRAFi-resistant cells. RNA-sequencing (RNA-seq) analysis identified altered expression patterns for several genes involved in various functional pathways in BRAFi-resistant cells compared with BRAFi-sensitive cells. Assay for transposase-accessible chromatin with high-throughput sequencing (ATAC-seq) was performed to measure alterations in chromatin accessibility, which revealed that chromatin accessibility was altered in both the promotor, in between and downstream regions of several genes. These changes resulted in alterations in the expression of several functional pathways, promoting resistance. An integrated analysis combining the RNA-seq and ATAC-seq datasets identified a group of genes for which changes in chromatin accessibility aligned with the changes in mRNA expression levels. Pathway analysis of these differentially expressed genes revealed the involvement of several oncogenic signaling pathways in the development of resistance to MAPK pathway inhibitors in melanoma cells. The present study describes the application of new and advanced approaches for identifying changes in chromatin accessibility that modulate gene expression and signaling pathway activities to promote MAPK pathway inhibitor resistance in *BRAF*-mutant melanoma cells.

## Materials and Methods

### Cell Culture

M229 parent and M229 BRAFi-resistant lines were gifts from Roger Lo, University of California, Los Angeles, and Neil Rosen, Memorial Sloan Kettering Cancer Center, New York. They were maintained in a humidified atmosphere of 5% CO_2_ at 37°C in Dulbecco’s modified Eagle medium (Life Technologies, Carlsbad, CA, USA) supplemented with 10% fetal bovine serum (Life Technologies) and 1% penicillin/streptomycin (Life Technologies).

### Cell Viability Assay (MTT Assay)

For MTT assays, 3 × 10^3^ melanoma cells (M229-Par and M229-Res) were plated in triplicate in a volume of 100 µL in 96-well plates. After 24 h, the cells were treated with different concentrations of inhibitors. Cell viability was evaluated 5 days after treatment. To measure cell viability, 20 µl 5 mg/mL MTT solution dissolved in 1× phosphate-buffered saline was added to each well of the 96-well plate and incubated for 2 h at 37°C. The MTT solution was then removed, and 100 µL dimethyl sulfoxide (DMSO) was added to each well. After the contents were mixed by pipetting, absorbance was measured at 590 nm and 630 nm using the Biotek Synergy MX Multi Format Microplate Reader (Biotek, Winooski, VT, USA). The average absorbance at 630 nm was subtracted from the average absorbance at 590 nm, and the growth rate was plotted relative to the growth rate of vehicle-treated cells.

### Soft-Agar Assay

**Figure 1:**
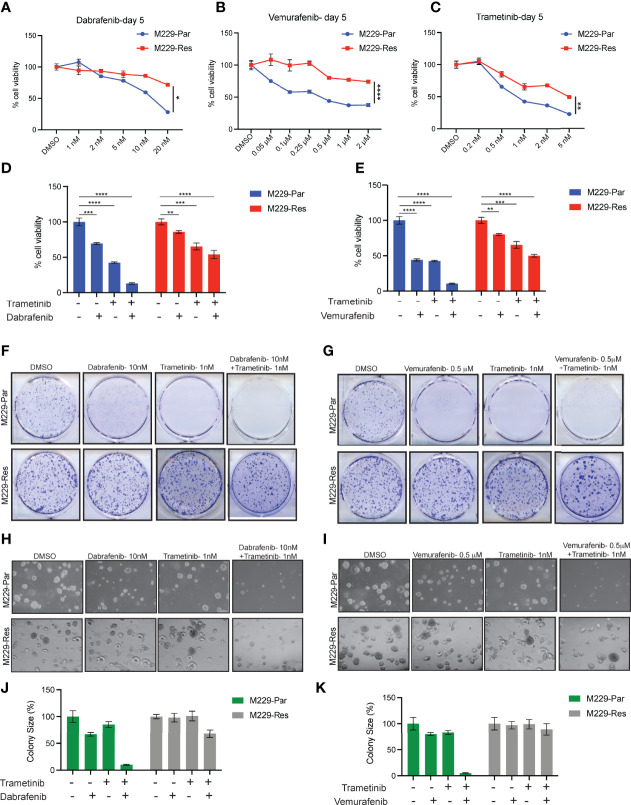
Effects of MAPK pathway inhibitors on M229-Par and M229-Res melanoma cell growth. **(A–C)** The indicated melanoma cell lines were treated with the indicated concentrations of dabrafenib, vemurafenib, or trametinib for 5 days and subjected to MTT assays. Cell viability is plotted relative to DMSO-treated cells. **(D)** The indicated melanoma cell lines were treated with either 10 nM dabrafenib or 1 nM trametinib, alone or in combination, for 5 days and subjected to MTT assays. Cell viability is plotted relative to DMSO-treated cells. **(E)** The indicated melanoma cell lines were treated with either 0.5 µM vemurafenib or 1 nM trametinib, alone or in combination, for 5 days and subjected to MTT assays. Cell viability is plotted relative to DMSO-treated cells. **(F)** The indicated melanoma cell lines were treated with either 10 nM dabrafenib or 1 nM trametinib, alone or in combination, and subjected to clonogenic assays. Representative images are shown. **(G)** The indicated melanoma cell lines were treated with either 0.5 µM vemurafenib or 1 nM trametinib, alone or in combination, and subjected to clonogenic assays. Representative images are shown. **(H)** The indicated melanoma cell lines were treated with either 10 nM dabrafenib or 1 nM trametinib, alone or in combination, and subjected to soft-agar assays. Representative images are shown. **(I)** The indicated melanoma cell lines were treated with either 0.5 µM vemurafenib or 1 nM trametinib, alone or in combination, and subjected to soft-agar assays. Representative images are shown. **(J–K)** Relative colony sizes in the images shown in panels **(H, I)**, respectively. Data represent the mean ± standard error of three biological replicates. ns = not significant, *p < 0.05, **p < 0.01, ***p < 0.001, ****p < 0.0001.

Soft-agar assays were performed by seeding 1 × 10^4^ melanoma cells (M229-Par and M229-Res) onto 0.4% low melting–point agarose (Sigma-Aldrich, Burlington, MA, USA) layered on top of 0.8% agarose. After 24 h, the cells were treated with different concentrations of inhibitors, as shown in the **Figure 1**, or DMSO (control). After 3–4 weeks of incubation, colonies were stained with 0.005% crystal violet and imaged under a microscope. Colony sizes were measured using ImageJ software (https://imagej.nih.gov/ij/) and plotted as percent relative colony size compared with control colonies. Statistical analysis was performed by Student’s *t*-test in GraphPad Prism 8.0 (GraphPad, San Diego, CA, USA).

### Clonogenic Assay

The clonogenic abilities of vehicle-treated (control) and inhibitor-treated melanoma cells (M229-Par and M229-Res) were measured in triplicate using clonogenic assays in which 0.5 × 10^3^ cells were seeded in a 6-well plate. After 24 h, the cells were treated with different concentrations of inhibitors, as shown in the **Figure 1**, or DMSO (control). After 1–2 weeks of treatment, colonies were fixed with a fixing solution containing 50% methanol and 10% acetic acid, followed by staining with 0.05% Coomassie blue (Sigma-Aldrich). The relative number of colonies was calculated by counting the number of colonies in each sample and plotting the average number of colonies.

### RNA-Sequencing

Total RNA was extracted from frozen cell pellet samples using the Qiagen RNeasy Plus Universal mini kit, according to the manufacturer’s instructions (Qiagen, Hilden, Germany). RNA samples were quantified using a Qubit 2.0 Fluorometer (Life Technologies), and RNA integrity was verified using an Agilent TapeStation 4200 (Agilent Technologies, Palo Alto, CA, USA). RNA-seq libraries were prepared using the NEBNext Ultra RNA Library Prep Kit for Illumina, according to the manufacturer’s instructions (NEB, Ipswich, MA, USA). Briefly, mRNAs were initially enriched with Oligo d(T) beads. Enriched mRNAs were fragmented for 15 minutes at 94°C. First-strand and second-strand cDNA were subsequently synthesized. cDNA fragments were end-repaired and adenylated at the 3’ends, and universal adapters were ligated to cDNA fragments, followed by index addition and library enrichment by PCR with limited cycles. The sequencing library was validated on the Agilent TapeStation and quantified using a Qubit 2.0 Fluorometer and by quantitative PCR (KAPA Biosystems, Wilmington, MA, USA).

The sequencing libraries were clustered on two lanes of a flowcell. After clustering, the flowcell was loaded on the Illumina HiSeq instrument (4000 or equivalent), according to the manufacturer’s instructions. The samples were sequenced using a 2 × 150-bp paired-end configuration. Image analysis and base calling were conducted using HiSeq Control Software. Raw sequence data (.bcl files) generated from Illumina HiSeq were converted into fastq files and de-multiplexed using Illumina bcl2fastq 2.17 software. One mismatch was allowed for index sequence identification.

### RNA-Sequencing Analysis

After investigating the quality of the raw data, sequence reads were trimmed to remove possible adapter sequences and nucleotides with poor quality using Trimmomatic v.0.36. The trimmed reads were mapped to the reference genome available on ENSEMBL using the STAR aligner v.2.5.2b. The STAR aligner uses a splice aligner that detects splice junctions and incorporates them to help align entire read sequences. BAM files were generated during this step. Unique gene hit counts were calculated using feature counts from the Subread package v.1.5.2. Only unique reads that fell within exon regions were counted.

Differentially expressed genes were identified using the DESeq2 program (17). Genes showing altered expression with p < 0.05 and fold change > 1.5 were considered to be differentially expressed. Goseq (18) was used to perform the gene ontology (GO) enrichment analysis, and Kobas was used to perform the KEGG pathway analysis (19).

### ATAC-Sequencing and Data Analysis

M229-Par and M229-Res cells were washed and treated with DNAse I (Life Tech, Cat. #EN0521) to remove genomic DNA contamination. Live cell samples were quantified and assessed for viability using a Countess Automated Cell Counter (ThermoFisher Scientific, Waltham, MA, USA). After cell lysis and cytosol removal, nuclei were treated with Tn5 enzyme (Illumina, Cat. #20034197) for 30 min at 37°C and purified with a MinElute PCR Purification Kit (Qiagen, Cat. #28004) to produce tagmented DNA samples. Tagmented DNA was barcoded with a Nextera Index Kit v2 (Illumina, Cat. #FC-131-2001) and amplified *via* PCR prior to an SPRI Bead cleanup to yield purified DNA libraries.

The reads were first mapped to the latest UCSC genome set using Bowtie2 version 2.1.0 (16). Mitochondrial reads, duplicate reads, and non-unique reads were removed before peak calling. MACS2 was used for peak calling using BAMPE mode (20). Differentially expressed peaks were identified using the DEseq2 program (17). Peaks showing altered expression with p < 0.05 and fold change > 1.5 were considered differentially expressed. Downstream genes of the differential peaks were used for GO and pathway enrichment analysis. Goseq (18) was used to perform the GO enrichment analysis, and Kobas was used to perform the KEGG pathway analysis (19).

### Integrated Analysis of RNA-Seq and ATAC-Seq Data

RNA-seq and ATAC-seq data were analyzed to identify same-direction changes in mRNA expression and chromatin accessibility. This integration was used to assess pathway enrichment using KEGG pathway analysis (KEGG; www.genome.jp/kegg/pathway.html).

### Statistical Analysis

All experiments were conducted in three biological replicates. The results for individual experiments were expressed as the mean ± SEM. P-values were calculated by t-test using GraphPad Prism version 8.0h for Macintosh, GraphPad Software, San Diego, California, USA (www.graphpad.com).

## Results

### Effects of MAPK Pathway Inhibitors on the Growth of BRAFi-Resistant and BRAFi-Sensitive Melanoma Cells

BRAFi-resistant M229 cells (M229-Res) were generated through the continuous exposure of BRAFi-sensitive M229 cells (M229-Par) to increasing concentrations of vemurafenib (PLX4032) *in vitro*, which allowed these cells to acquire a resistant phenotype (21). To determine whether the M229-Res cells generated by continuous vemurafenib exposure were also resistant to more potent and stronger BRAFi, such as dabrafenib, or to MEKi, such as trametinib, we performed short-term 3-(4,5-dimethylthiazol-2-yl)-2,5-diphenyltetrazolium bromide (MTT) survival assays. Vemurafenib is a potent inhibitor of B-Raf^V600E^, with a half-maximal inhibitory concentration (IC_50_) of 31 nM in a cell-free assay (22). Vemurafenib shows a 10-fold increase in selectivity for B-Raf^V600E^ compared with wild-type B-Raf in enzymatic assays, and cellular selectivity can exceed 100-fold for B-Raf^V600E^ compared with wild-type B-Raf (23). Dabrafenib is also a specific inhibitor of BRAFV600 mutants, with an IC_50_ of 0.7 nM in cell-free assays, and presents with 7- and 9-fold less potency against wild-type B-Raf and c-Raf, respectively (24). Dabrafenib is approximately 50 times more effective than vemurafenib for inhibiting B-Raf^V600E^ (25). Trametinib is a highly specific and potent MEK1/2 inhibitor, with an IC_50_ ranging from 0.92 to 1.8 nM in cell-free assays and displaying no inhibitory effects against the kinase activities of c-Raf, B-Raf, or ERK1/2 (26–28). Using the MTT assay, we found that the inhibition of the BRAF→MEK→ERK pathway by vemurafenib, dabrafenib, or trametinib resulted in stronger effects in M229-Par cells than in M229-Res cells (**Figures 1A–C**). We also observed that BRAFi and MEKi when used in combination, strongly inhibited the growth of M229-Par cells with modest effects on M229-Res cells (**Figures 1D, E**). Combined BRAFi and MEKi treatment also strongly inhibited the growth of M229-Par cells compared with M229-Res cells in clonogenic (**Figures 1F, G**) and soft-agar assays (**Figures 1H–K**). These results indicate that M229-Res cells generated by continuous exposure to vemurafenib also developed resistance against the more potent BRAFV600 inhibitor dabrafenib and the MEK1/2 inhibitor trametinib, both alone and in combination.

### RNA-Sequencing Analysis Identifies Alterations in the Transcriptome of BRAFi-Resistant Relative to BRAFi-Sensitive Melanoma Cells

**Figure 2:**
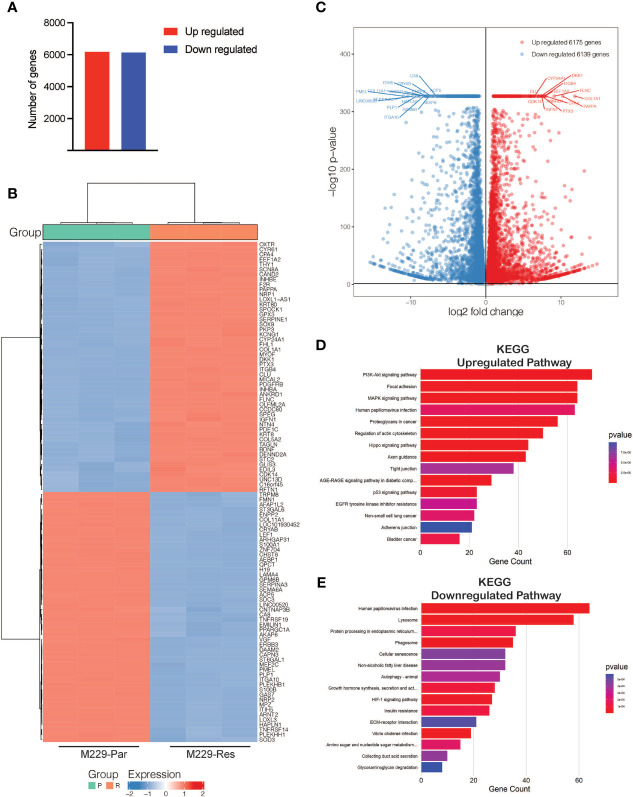
RNA-sequencing identified differentially expressed mRNAs between M229-Par and M229-Res cells. **(A)** Total number of upregulated or downregulated genes with adjusted *p*-values ≤ 0.05 in M229-Res compared with M229-Par samples. **(B)** Heat map showing differentially expressed genes (up- or downregulated) in the indicated comparisons. The top 50 upregulated and the top 50 downregulated genes based on p-values are shown. **(C)** Volcano plot showing differentially expressed genes (up- or downregulated) in the indicated comparisons. The top 15 upregulated and the top 15 downregulated genes based on p-values are also labeled. **(D, E)** KEGG pathway analysis showing key upregulated **(D)** and downregulated **(E)** biological pathways associated with differentially expressed mRNAs in M229-Res cells compared with M229-Par cells.

To identify transcriptional changes in *BRAF*-mutant M229 BRAFi-resistant cells as compared to BRAFi-sensitive cells, we performed RNA-seq comparing M229-Res cells with M229-Par cells. RNA-seq identified 12,314 differentially expressed genes (adjusted *p*-values ≤ 0.05), including 6,139 downregulated and 6,175 upregulated genes (**Figure 2A**; **Supplementary Table S1**) in M229-Res cells compared with M229-Par cells. A heat map showing the top 100 affected genes (50 upregulated and 50 downregulated) and a volcano plot showing the top 30 genes (15 upregulated and 15 downregulated), based on the log_2_ fold change values, were plotted (**Figures 2B, C**).

To explore the functional pathways likely to be activated or repressed by changes in gene expression, we performed functional pathway analysis using the Kyoto Encyclopedia of Genes and Genomes (KEGG). Our results showed that genes upregulated in M229-Res cells compared with M229-Par cells were associated with the activation of many oncogenic pathways, including phosphoinositide 3-kinase (PI3K)–protein kinase B (AKT), MAPK, focal adhesion, and proteoglycan-based signaling cascades (**Figure 2D**). Additionally, the identified downregulated genes were associated with the inhibition of anti-proliferative pathways, such as cellular senescence, autophagy, and glycosaminoglycan degradation (**Figure 2E**). Our results demonstrate that transcriptional changes in M229-Res cells promote the activation of pro-oncogenic signaling pathways and the inhibition of anti-cancer pathways.

### ATAC-Sequencing Analysis Identifies Alterations in Chromatin Accessibility Regions in BRAFi-Resistant Compared With BRAFi-Sensitive Melanoma Cells

**Figure 3:**
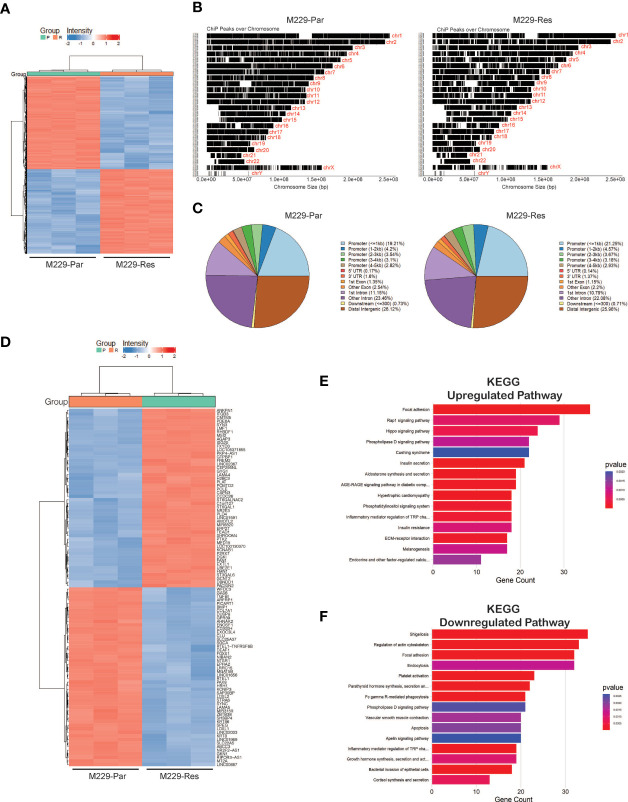
ATAC-sequencing identified differentially expressed mRNAs between M229-Par and M229-Res cells. **(A)** Heatmaps showing differential genomic regions with increased or decreased chromatin accessibility based on ATAC-seq in M229-Res compared with M229-Par samples. **(B)** Chromatin-immunoprecipitation (ChiP) peaks over chromosomes analyzed by ATAC-seq in M229-Res and M229-Par samples. **(C)** Pie-chart for the indicated samples mapping the locations of annotated peaks identified by ATAC-seq. **(D)** Heat map showing the top 50 upregulated and the top 50 downregulated genes with increased and decreased chromatin accessibility based on p-values. **(E, F)** KEGG pathway analysis showing key upregulated **(E)** and downregulated **(F)** biological pathways associated with genes located in regions with increased or decreased chromatin accessibility in M229-Res cells compared with M229-Par cells.

Open chromatin accessible regions contain cis-regulatory elements that might modulate gene expression and activity (29, 30). We, therefore, performed chromatin accessibility profiling analyses in both M229-Par and M229-Res cells using ATAC-seq. We identified a total of 71,542 (adjusted *p*-values ≤ 0.05) accessible regions in M229-Res cells compared with M229-Par cells, including 37,814 regions with negative chromatin accessibility and 33,728 regions with positive chromatin accessibility (**Figures 3A, B**; **Supplementary Table S2**). Altered chromatin accessibility regions were distributed across upstream, intergenic, and downstream regions (**Figure 3C**). A heat map showing the top 100 genes (50 upregulated and 50 downregulated) due to changes in chromatin accessibility was plotted (**Figure 3D**). To understand how changes in chromatin accessibility impact the regulation of functional pathways involved in the development of resistance to MAPK pathway inhibitors in M229 cells, we performed functional pathways analysis using KEGG. Our results showed that changes in chromatin accessibility upregulated the expression of genes involved in several oncogenic pathways, such as the Rap1, Hippo, and extracellular matrix receptor interaction–dependent signaling cascades (**Figure 3E**). Apoptosis, among other pathways, was downregulated in M229-Res cells compared with M229-Par cells (**Figure 3F**). These results demonstrate that M229-Res cells display a unique chromatin accessibility profile that supports the development of MAPK pathway inhibitor resistance.

### Integrated ATAC-Sequencing and RNA-Sequencing Data Analysis Identifies Alterations in Chromatin Accessibility Regions That Align With Changes in mRNA Expression Level in BRAFi-Resistant Compared With BRAFi-Sensitive Melanoma Cells

**Figure 4:**
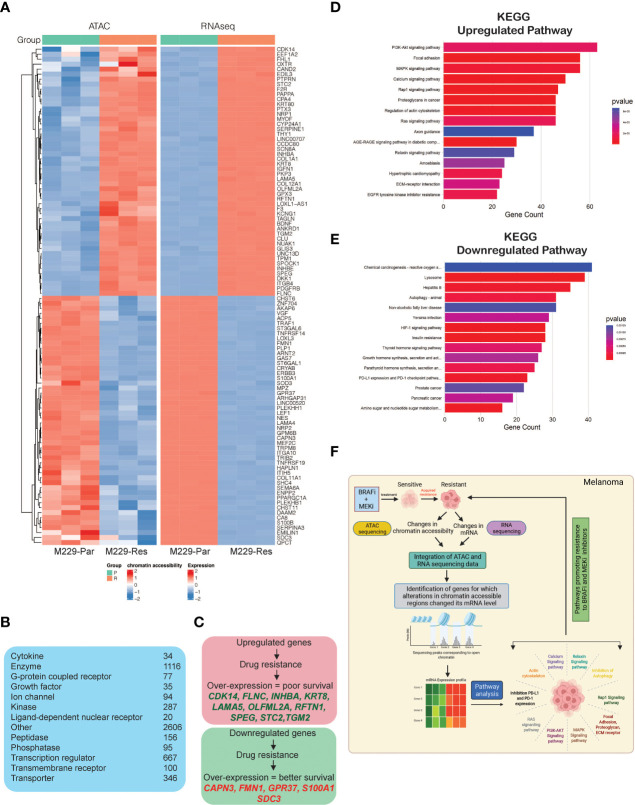
Integrated analysis of ATAC-sequencing and RNA-sequencing to identify differentially expressed genes between M229-Par and M229-Res cells for which changes in mRNA expression aligned with changes in chromatin accessibility. **(A)** Heatmap for the top 100 (50 upregulated or with increased chromatin accessibility and 50 downregulated or with reduced chromatin accessibility) genes showing similar patterns in both the ATAC-seq and RNA-seq analyses in M229-Res cells compared with M229-Par cells. **(B)** Genes obtained integrated from analysis categorized based on function. **(C)** Survival analysis (3-year) for patients with melanoma according to high and low expression levels of genes showing similar patterns in both the ATAC-seq and RNA-seq analyses in M229-Res cells compared with M229-Par cells using the Human Protein Atlas dataset. **(D, E)** KEGG pathway analysis showing key upregulated **(D)** and downregulated **(E)** biological pathways associated with genes that display coherence between changes in chromatin accessibility and changes in mRNA expression levels in M229-Res cells compared with M229-Par cells. **(F)** Model: ATAC-seq integrated with RNA-seq is a new and advanced approach for identifying unique changes in chromatin accessibility regions that modulate gene expression and signaling pathway activities to promote the MAPK pathway inhibitor resistance.

To identify correlations between accessible chromatin regions and mRNA expression levels, we integrated the data obtained from ATAC-seq and RNA-seq analyses, which revealed 5,646 significant changes in chromatin accessibility (adjusted *p*-values ≤ 0.05) aligned with significant changes in mRNA expression levels (adjusted *p*-values ≤ 0.05). Chromatin accessibility increased for 2,038 regions, associated with the upregulation of mRNA expression, whereas 3,608 chromatin regions became less accessible, inhibiting mRNA expression (**Supplementary Table S3**). A heat map was plotted to display the top 100 genes (50 upregulated and 50 downregulated) based on the log_2_ fold changes in mRNA expression among those aligned with changes in chromatin accessibility (**Figure 4A**). These included transcriptional regulators, ion channels, enzymes, kinases, phosphatases, growth factors, G-protein coupled receptors and transmembrane receptors (**Figure 4B**). A subset of top upregulated and downregulated genes owing to their association with the pathways involved in promoting MAPK pathway inhibitor resistance and treatment failure, predicted 3-year survival outcomes among melanoma patients (**Figure 4C**).

To identify which functional pathways were affected by these gene expression changes, we performed KEGG pathway analysis (**Supplementary Table S3**) and identified several key biological processes enriched in M229-Res cells compared with M229-Par cells (**Figures 4D, E**), which may be involved in promoting a treatment-resistant phenotype. In particular, we observed the significant upregulation of PI3K–AKT, MAPK, Rap1, Ras, and proteoglycan, actin cytoskeleton and ECM receptor interaction dependent signaling pathways in M229-Res cells compared with M229-Par cells (**Figure 4D**). We also observed the inhibition of tumor growth inhibitory pathways, such as autophagy and the programmed death-ligand 1 (PD-L1) and programmed cell death protein 1 (PD-1) checkpoint pathway (**Figure 4E**). These results suggest that changes in the global chromatin state in M229-Res cells lead to altered gene expression associated with biological pathways that promote MAPK pathway inhibitor resistance in *BRAF*-mutant melanoma cells. Additionally, the expression of many genes predicted survival among melanoma patients.

## Discussion

BRAF is a member of the Raf kinase family, and the oncogenic V600E mutation in *BRAF* has been identified in 90% of melanoma cases, leading to the activation of the MAPK pathway (31–33). Several oncogenic BRAF-targeting inhibitors have been approved by the US FDA, including vemurafenib and dabrafenib, for the clinical treatment of metastatic melanoma (8, 34, 35). Although BRAFi therapy results in an impressive initial clinical response against *BRAF*-mutant metastatic melanoma, the durability of this response is limited by the rapid emergence of acquired BRAFi resistance, which often occurs within a few months of treatment initiation (36–39). In the clinic, BRAFi therapy is often combined with other MAPK pathway inhibitors, such as MEKi, to obtain durable effects for the suppression of melanoma growth and the avoidance of drug resistance (6, 10, 12). However, acquired resistance to these agents remains a major hurdle preventing the success of targeted therapies and limiting their benefits. One approach to overcoming this limitation is to understand the mechanisms underlying acquired resistance (40–42), which can contribute to modifying therapeutic regimens or developing combination therapies to prevent the emergence of drug resistance.

In this study, we performed a large-scale, deep-sequencing analysis to investigate reprogramming of chromatin landscape, a MAP kinase pathway independent mechanism in acquired MAPK pathway inhibitor resistance in melanoma. Our results are summarized in **Figure 4F**. We found that melanoma cells that are resistant to the BRAFi vemurafenib are also resistant to the more potent BRAFi dabrafenib and to combination treatment including both BRAFi (vemurafenib and dabrafenib) and MEKi (trametinib). Previous studies have shown that cancer cells have distinct genetic, epigenetic and transcriptional states, which allows them to exist in heterogeneous functional populations and that poses a major obstacle to cancer diagnosis and treatment (43). To identify the differential chromatin state, MAPK pathway inhibitor–resistant and sensitive melanoma cells were analyzed using high-throughput sequencing methods. Analysis of the results let us to discover global changes in chromatin accessibility regions located upstream, in between, and downstream of numerous genes, resulting in changes in mRNA expression. These genes that were enriched in MAPK pathway inhibitor–resistant melanoma cells belonged to distinct functional classes which includes kinases, phosphatases, transcription regulator, transporters, growth factor, enzyme, g-protein coupled receptors. Previous studies have provided the evidence that kinases (44, 45), phosphatases (46), transcription regulator (47), transporters (48), growth factor (49, 50), enzymes (51), g-protein coupled receptors (52) drive drug resistance in cancer and provides the opportunity for targeting them to combat drug resistance. Additionally, the expression levels of many of these altered genes were able to predict survival in melanoma patients. Thus, in the future, these candidate genes may serve as biomarkers that can predict the subgroup of patients able to benefit from MAPK pathway inhibitor therapy, identify patients who will develop resistance to MAPK pathway inhibitors early on and select the optimal therapeutic approaches for treating these patients.

Pathway analysis performed with the genes identified as potentially upregulated due to changes in chromatin accessibility were associated with several known oncogenic signaling pathways involved in tumor growth, metastasis, and cancer drug resistance, such as Ras signaling (21, 53), the MAPK pathway (12, 54), and PI3–AKT signaling (55, 56). In addition to known tumor-promoting signaling cascades, our study identified pathways that never been investigated for their role in promoting resistance to MAPK pathway inhibitors in melanoma, such as relaxin, calcium, and Rap1 signaling. Additionally, our study identified many genes that were downregulated due to closed chromatin, associated with the suppression of anti-growth signaling pathways, such as senescence and apoptosis.

Apart from genes that changed at both chromatin accessibility level and transcription level, there were candidates that only changed at the mRNA level. This was because of the limitation of these two methods. Fundamentally, the transcriptome measured *via* RNA seq is the result of transcription, posttranscriptional regulation and RNA degradation, while the chromatin accessibility changes measured *via* ATAC seq provides information on chromatin accessibility across the genome that effect transcriptional initiation site availability at that particular time point. Additionally, the probability of ATAC-seq to not accurately predict all the changes in chromatin accessibility also exist. These are few limitations of using ATAC seq and RNA seq together to identify the genes that are altered in drug resistant state as compared to the sensitive state and has to be considered while concluding the results. Although important, to resolve this issue is beyond the current scope of our manuscript.

In sum, our results suggest that the development of drug resistance in cancer cells is a complex process. Hence, a deeper understanding of these newly identified mechanisms will provide better insights into the development of MAPK pathway inhibitor resistance in melanoma and potentially lead to more efficient treatment options. These studies also lay the foundation for further examinations of newly identified genes and pathways involved in the development of resistance to MAPK pathway inhibitors in our study in preclinical mouse models of melanoma. Collectively, our study results provide insight into the comprehensive changes in chromatin accessibility changes that regulate the transcriptional outputs and signaling cascades to promote resistance to MAPK pathway inhibitors in melanoma. Our study also identified new biomarkers, targets, and signaling pathways that can be investigated to formulate new melanoma treatments, particularly for patients who have developed resistance to MAPK pathway inhibitors (BRAFi + MEKi).

## Data Availability Statement

The datasets presented in this study - the RNA-seq and ATAC-seq are deposited with accession number GSE202122 (GEO accession# GSE202118 for RNA-seq and GSE202117 for ATAC-seq) and can be found in online repository geo@ncbi.nlm.noh.gov.

## Author Contributions

RG conceived the research and designed the experiments. KR and PG performed the experiments. YE helped with bioinformatics analysis. RG interpreted the data and wrote the manuscript. All authors reviewed, made suggestions, approved the paper, and provided comments.

## Funding

We gratefully acknowledge grants from the National Institutes of Health (NIH) [R03CA221926 (to RG), R03CA230815 (to RG), R03CA248913 (to RG), and R01CA233481 (to RG)].

## Conflict of Interest

The authors declare that the research was conducted in the absence of any commercial or financial relationships that could be construed as a potential conflict of interest.

## Publisher’s Note

All claims expressed in this article are solely those of the authors and do not necessarily represent those of their affiliated organizations, or those of the publisher, the editors and the reviewers. Any product that may be evaluated in this article, or claim that may be made by its manufacturer, is not guaranteed or endorsed by the publisher.

## Supplementary Material

Supplementary Table 1 All genes differentially expressed genes (adjusted p-values ≤ 0.05) in M229-Par and M229-Res cells identified via RNA sequencing.

Supplementary Table 2 All significantly chromatin accessible regions altered with (adjusted p-values ≤ 0.05) in M229-Par and M229-Res cells identified via ATAC sequencing.

Supplementary Table 3 Integration of ATAC sequencing and RNA sequencing data with significantly altered chromatin accessible regions aligning with gene expression changes in in M229-Par and M229-Res cells.
